# Editorial: Achieving Effective Management and Treatment of Diabetes Mellitus in Future Primary Care

**DOI:** 10.3389/fendo.2022.854244

**Published:** 2022-03-17

**Authors:** Indah Suci Widyahening, Kamlesh Khunti, Rimke C. Vos, Boon-How Chew

**Affiliations:** ^1^Department of Community Medicine, Faculty of Medicine Universitas Indonesia, Jakarta, Indonesia; ^2^National Institute for Health Research Applied Research Collaboration-East Midlands, Leicester Diabetes Centre, Leicester, United Kingdom; ^3^Diabetes Research Centre, Leicester General Hospital, University of Leicester, Leicester, United Kingdom; ^4^Department Public Health and Primary Care/Leiden University Medical Center (LUMC)-Campus The Hague, Leiden University Medical Center, The Hague, Netherlands; ^5^Department of Family Medicine, Faculty of Medicine and Health Sciences, Universiti Putra Malaysia (UPM), Serdang, Malaysia; ^6^Clinical Research Unit, Hospital Pengajar Universiti Putra Malaysia (HPUPM Teaching Hospital), Persiaran Mardi - UPM (University Putra Malaysia), Serdang, Malaysia

**Keywords:** diabetes, primary care, treatment, quality of care, comprehensive management

Currently, 537 million adults aged 20–79 years have diabetes mellitus (DM) worldwide. It has become one of the fastest-growing health challenges of the 21st century, with a disproportionate burden on low and middle-income countries (1). Considering the significant burden of the disease and its complications, tackling DM at the primary care level becomes imperative. Within the health care system, primary care plays a central role by providing diabetes care in the community for the majority of people with type 2 diabetes (T2D) while forging necessary integrated collaborative care with secondary or tertiary health care providers of multidisciplinary professionals (2). For most people with T2D, primary care physicians are the first point of contact. There is strong evidence worldwide that primary diabetes care can provide cost-effective, comprehensive, and patient-centered management to prevent and treat T2D and its related conditions. For people with type 1 diabetes (T1D), although a specialist diabetes team usually manages them, the involvement of primary care in delivering aspects of care such as monitoring and managing secondary complications is increasing.

The cornerstone of managing T2D is promoting lifestyle interventions that include a healthy diet and regular exercise to attain and maintain healthy body weight. Pharmacological interventions include controlling cardiovascular risk factors such as smoking cessation, blood pressure, and blood cholesterol to personalized and safe targets. Physical exercise plays an essential role as a non-pharmacological and cost-effective treatment, improves insulin sensitivity, a better quality of life (QoL), enhances diabetes treatment efficacy, thus lowering morbidity and mortality in people with T2D. Many types of physical exercise have been studied for their effectiveness in the management of DM, including low to moderate intensity ‘traditional’ mind-body exercises such as Tai Chi. According to the systematic review by Qin et al. included in this Research Topic, Tai Chi improved QoL and decreased BMI for people with T2D when compared to either wait-list, no intervention, usual care or sham exercise. Despite its proven benefit, encouraging physical activity among people with (T1D) remains challenging. Undertaking physical exercise needs to be balanced with insulin dosing and food intake to maintain safe blood glucose levels before, during, and after exercise. Participation in a clinical trial on exercise with a multimodal approach which incorporated education support and an interstitial glucose monitoring system improve nocturnal glycemia significantly and reduce insulin use in people with T1D, as reported by McCarthy et al. Self-management skills are essential in fostering lifestyle changes to achieve good metabolic control among people with diabetes. Diabetes self-management education is considered one of the pillars in T2D care. In a randomized controlled trial, van der Velde et al. report that a self-management education program named the “Beyond Good Intentions” (BGI) effectively improved the dietary quality among people with T2D compared to usual care.

T2D and its complications are preventable by ensuring continuous access to prompt and well-organized care, structured patient education, and optimized risk factor control. Several innovative models of care that could enhance T2D management within primary care have been developed and studied. Pay-for-performance (P4P) was introduced as a strategy to improve healthcare quality through financial incentives. Lian et al. provide evidence that implementing a P4P Program to usual care could lower the risk of depression among T2D in Taiwan. In Canada, Hersson-Edery et al. explore the feasibility of a Diabetes Empowerment Group Program (DEGP) to foster patient engagement and empowerment in diabetes self-care and identified seven critical elements: medical visit, continuity of care, group-based dynamics, multi-disciplinarity, clinician facilitation, patient-centered agenda, and a theoretical framework of empowerment The theoretical framework itself comprises of four components: attitude, knowledge, behavior, and relatedness. This goes well with the implementation of shared medical appointments (SMAs) in the management of T2D. SMAs is defined as “consecutive individual medical visits carried out in a supportive group setting of patients of similar medical conditions where all can listen, interact, and learn” (3). Ee et al. report their experience in piloting SMAs in primary care for people with T2DM in Western Sydney, Australia. They found that SMAs, which included a structured education program and mindfulness component, was feasible and acceptable and resulted in lower total cholesterol levels and pain intensity.

T2D is a progressive disease; hence many people with T2D will require insulin therapy during the course of their illness. However, psychological opposition towards insulin use, known as “psychological insulin resistance” (PIR), is frequently found in people with T2DM and healthcare providers (4). The findings of a cross-sectional study by Boels et al. may enhance discussion of this problem as they found that those on insulin therapy have worse vitality, general health, and barriers to activity compared to those receiving oral antihyperglycemic agents only.

In recent years, the armamentarium for the management of hyperglycemia has been strengthened by the introduction of novel therapies, including sodium-glucose cotransporter 2 (SGLT2) inhibitors and Glucagon-Like Peptide-1 (GLP-1) Receptor Agonists. The cardiovascular and kidney protective effects of SGLT2 inhibitors and GLP-1 receptor agonists have now been ascertained, independent of their glucose-lowering properties, and are now recommended as first-line therapies in people with atherosclerotic cardiovascular disease, heart failure, and chronic conditions kidney disease. To add to the evidence, Wei et al., in their meta-analysis of randomized controlled trials, found that SGLT2 inhibitors can remarkably reduce hepatic enzymes, hepatic fat and improve body composition in T2D patients with non-alcoholic fatty liver disease (NAFLD). Thus, providing a new treatment option for this group of patients. Meanwhile, Kim et al. conducted a network meta-analysis to evaluate the effect and safety of adding oral hypoglycemic agents (metformin, SGLT2 inhibitor, or SGLT1/2 co-inhibitor) or injectable GLP-1 RAs to insulin therapy in patients with T1D. They suggest that sotagliflozin and short-acting GLP-1RAs as add-on therapies could have beneficial effects in lowering the HbA1c level, insulin dose, and body weight in patients with T1D.

There is growing evidence suggesting that vitamin D deficiency could play an essential role in T2D pathogenesis (5). Thus, the potential of vitamin D supplementation as part of the management of T2DM to optimize glycemic control and prevent complications were investigated in a randomized controlled trial by Cojic et al. They found that oral daily doses of vitamin D significantly decreased the level of HbA1c after 3 and 6 months of vitamin D supplementation with metformin, compared to the metformin only group. However, the effect of vitamin D on insulin resistant index (calculated as homeostatic model assessment; HOMA-IR), and oxidative stress markers (measured as malondialdehyde levels and Tryglicerides/Thiobarbituric acid-reactive substances (TG/TBARS) index), was not statistically significant.

Diabetes is one chronic condition where the evidence is changing at a pace. The collection of studies presented in this Research Topic of Frontiers in Endocrinology has further advanced the evidence for DM management in primary care.

## Author Contributions

IW prepared the original draft. KK, RV, and B-HC critically review and edit the manuscript. All authors have reviewed and approved of the final manuscript.

## Funding

KK is supported by the National Institute for Health Research (NIHR) Applied Research Collaboration East Midlands (ARC EM) and the NIHR Leicester Biomedical Research Centre (BRC).

## Conflict of Interest

KK has acted as a consultant, speaker or received grants for investigator-initiated studies for Astra Zeneca, Novartis, Novo Nordisk, Sanofi-Aventis, Lilly and Merck Sharp & Dohme, Boehringer Ingelheim, Bayer, Berlin-Chemie AG/Menarini Group, Janssen, and Napp.

The remaining authors declare that the research was conducted in the absence of any commercial or financial relationships that could be construed as a potential conflict of interest.

## Publisher’s Note

All claims expressed in this article are solely those of the authors and do not necessarily represent those of their affiliated organizations, or those of the publisher, the editors and the reviewers. Any product that may be evaluated in this article, or claim that may be made by its manufacturer, is not guaranteed or endorsed by the publisher.
